# Type IV Collagen Variants in CKD: Performance of Computational Predictions for Identifying Pathogenic Variants

**DOI:** 10.1016/j.xkme.2020.12.007

**Published:** 2021-02-10

**Authors:** Cole Shulman, Emerald Liang, Misato Kamura, Khalil Udwan, Tony Yao, Daniel Cattran, Heather Reich, Michelle Hladunewich, York Pei, Judy Savige, Andrew D. Paterson, Mary Ann Suico, Hirofumi Kai, Moumita Barua

**Affiliations:** 1 Division of Nephrology, University Health Network, Toronto, Canada; 2 Toronto General Hospital Research Institute, Toronto General Hospital, Toronto, Canada; 3 Department of Molecular Medicine, Graduate School of Pharmaceutical Science, Kumamoto University, Kumamoto, Japan; 4 Institute of Medical Sciences, Toronto, Canada; 5 Department of Medicine, Toronto, Canada; 6 University of Melbourne, Melbourne, Australia; 7 Division of Epidemiology and Biostatistics, Dalla Lana School of Public Health, Toronto, Canada; 8 Genetics and Genome Biology, Research Institute at Hospital for Sick Children, Toronto, Canada

**Keywords:** Alport syndrome, FSGS, type IV collagen variants, genomics, in silico predictions, computational predictions, ClinVar, ARUP, LOVD, gnomAD

## Abstract

**Rationale & Objective:**

Pathogenic variants in type IV collagen have been reported to account for a significant proportion of chronic kidney disease. Accordingly, genetic testing is increasingly used to diagnose kidney diseases, but testing also may reveal rare missense variants that are of uncertain clinical significance. To aid in interpretation, computational prediction (called in silico) programs may be used to predict whether a variant is clinically important. We evaluate the performance of in silico programs for *COL4A3/A4/A5* variants.

**Study Design, Setting, & Participants:**

Rare missense variants in *COL4A3/A4/A5* were identified in disease cohorts, including a local focal segmental glomerulosclerosis (FSGS) cohort and publicly available disease databases, in which they are categorized as pathogenic or benign based on clinical criteria.

**Tests Compared & Outcomes:**

All rare missense variants identified in the 4 disease cohorts were subjected to in silico predictions using 12 different programs. Comparisons between the predictions were compared with: (1) variant classification (pathogenic or benign) in the cohorts and (2) functional characterization in a randomly selected smaller number (17) of pathogenic or uncertain significance variants obtained from the local FSGS cohort.

**Results:**

In silico predictions correctly classified 75% to 97% of pathogenic and 57% to 100% of benign *COL4A3/A4/A5* variants in public disease databases. The congruency of in silico predictions was similar for variants categorized as pathogenic and benign, with the exception of benign *COL4A5* variants, in which disease effects were overestimated. By contrast, in silico predictions and functional characterization classified all 9 pathogenic *COL4A3/A4/A5* variants correctly that were obtained from a local FSGS cohort. However, these programs also overestimated the effects of genomic variants of uncertain significance when compared with functional characterization. Each of the 12 in silico programs used yielded similar results.

**Limitations:**

Overestimation of in silico program sensitivity given that they may have been used in the categorization of variants labeled as pathogenic in disease repositories.

**Conclusions:**

Our results suggest that in silico predictions are sensitive but not specific to assign *COL4A3/A4/A5* variant pathogenicity, with misclassification of benign variants and variants of uncertain significance. Thus, we do not recommend in silico programs but instead recommend pursuing more objective levels of evidence suggested by medical genetics guidelines.

**Plain-Language Summary**

Type IV collagen mutations have been reported to account for a significant proportion of chronic kidney disease. As a result, genetic testing is increasingly being used for diagnosis but can uncover DNA changes that are of uncertain clinical significance. To determine whether causative for disease (called pathogenic), DNA changes can be tested with cell and animal models, an approach that is limited by the absence of well-established models for most genes, expense, and time-consuming nature. Alternatively, computational programs can be used to make predictions for pathogenicity. In this report, we begin to define the test characteristics for these computational predictions using bioinformatic and experimental approaches, with results suggesting that programs tend to overestimate the effects of DNA changes.

Chronic kidney disease (CKD) represents a heterogeneous group of disorders that result in irreversible fibrosis over time. Current diagnostic methods often fail to distinguish molecular mechanisms or predict disease course. CKD affects more than 750 million people globally and results in more than 1 million deaths annually. As such, kidney disease is a major health burden with substantive costs.^1^^,^^2^

Genomics is emerging as one tool to identify mechanistically relevant CKD subtypes. Using whole-exome sequencing, we have recently reported that pathogenic variants in the *COL4A3*/*A4*/*A5* genes are the leading single gene causes (∼5%) of focal and segmental glomerulosclerosis (FSGS), a histopathologic entity representing diverse causes.^3^ Similarly, pathogenic variants in the *COL4A3*/*A4*/*A5* genes have also been reported to account for a significant proportion of CKD.^4^ Pathogenic variants in type IV collagen are well known to cause Alport syndrome.^5^, ^6^, ^7^, ^8^, ^9^, ^10^

The human genome has tremendous sequence variation and the effect of rare nonsynonymous single-nucleotide variants (SNVs) in a disease-associated gene can be unclear. The American College of Medical Genetics (ACMG) has standards based on expert consensus for declaring the pathogenicity of rare variants that are organized into supporting, moderate, strong, and very strong levels of evidence.^11^ Some of these criteria include assessment of frequency in population data, type of variant change (eg, null variant), identification of familial cosegregation, presence in clinically ascertained mutation databases, bioinformatics, and functional data.^12^^,^ Well-established functional studies that show a deleterious effect are considered strong levels of evidence.^13^

Computational predictions, also known as in silico programs, are one part of clinical variant classification in the diagnostic setting but are considered supportive compared with stronger lines of evidence. These programs have been developed to predict the functional effects of rare missense variants. Broadly, the algorithms use different types of variant information, including sequence conservation, protein structure analysis, and meta prediction (using results from multiple programs) for predictions.^14^, ^15^, ^16^, ^17^, ^18^

The predictive performance of in silico programs has been evaluated with computational methods against data sets that contain pathogenic and benign variants obtained from public resources (eg, Universal Protein Resource [Uniprot]), literature, and curated disease databases in which variants in kidney disease genes are not highly represented.^19^, ^20^, ^21^, ^22^, ^23^, ^24^, ^25^, ^26^ We evaluate the predictive performance of in silico programs for *COL4A3*/*A4*/*A5* missense variants by first comparing with clinically categorized variants deposited in 3 public disease databases and a local FSGS cohort. As a second approach, in silico predictions are compared with functionally characterized missense variants identified in the local FSGS cohort.

## Methods

### Whole-Exome Sequencing Analysis

Details on how patients were recruited and exome data analyzed have been previously described.^3^ Study participants gave their written informed consent and the study protocol was approved by the Toronto General Hospital’s committee on human research (98-UO13). Whole-exome sequencing and data processing were performed by The Centre for Applied Genomics, The Hospital for Sick Children, Toronto, Canada. Exomic capture was achieved with Agilent SureSelect Human All Exon V5. Reads were mapped to the hg19 reference sequence.

### Variant Calling From FSGS Whole-Exome Sequencing Data

Variants were identified using GATK (version 4.0.5.1).^27^ Gene-based annotation features of ANNOVAR were applied (access date, April 16, 2018).^28^ The frequency of variants was determined using Genome Aggregation Database (gnomAD; version 2.1.1; access date, March 18, 2019).^26^^,^^29^, ^30^, ^31^ Variants in *COL4A3*/*A4*/*A5* were categorized as rare if having a minor allele frequency ≤ 0.005 in the ethnically matched population within gnomAD. This cutoff was selected in consideration of the low prevalence of FSGS, estimated at 7 per million for the general population, 20 per million for Africans, and 5 per million for Europeans.^32^^,^^33^ It was also selected in consideration of inheritance patterns: *COL4A3*/*A4*/*A5* is associated with autosomal recessive, dominant, or X-linked recessive disease. Rare missense variants in *COL4A3*/*A4*/*A5* were designated as pathogenic if reported in other cases of kidney disease after searching the literature and disease databases ClinVar, ARUP, and LOVD.^3^^,^^34^

### In Silico Predictions Programs

Rare *COL4A3*/*A4*/*A5* missense variants from our FSGS whole-exome sequencing data and disease databases ClinVar, ARUP, and LOVD (accessed October 22, 2019, September 13, 2019, and August 28, 2019, respectively) were identified. All rare SNVs reported in these sources have already been categorized. We in turn submitted the missense variants to 12 in silico programs for predictions (Table S1).^15^^,^^24^^,^^35^, ^36^, ^37^, ^38^, ^39^^,^^40^, ^41^, ^42^, ^43^, ^44^, ^45^^,^^46^ A variant was categorized as pathogenic if the majority, selected as 10 or more of 12 programs, categorized the variant as pathogenic using the program’s recommended scoring cutoffs.

### *COL4A* Split Luciferase Assay

From our FSGS cohort with whole-exome sequencing data, 9 pathogenic variants and 8 variants of uncertain significance in *COL4A3* and *COL4A5* were randomly selected. We defined pathogenic variants as rare (minor allele frequency < 0.005) and reported in other cases with kidney disease, whereas variants of uncertain significance were defined as any other rare missense variant. To assess heterotrimer formation ability of these missense variants, we used the split complementation Nano-luciferase assay system that we have previously developed.^47^ Tagged plasmid constructs of *COL4A4*-FLAG, wild-type or mutant *COL4A3*-SmBiT, and *COL4A5*-LgBiT were generated as described previously.^47^ Corresponding SmBiT and LgBiT tags were attached at either the N-terminal or C-terminal of *COL4A3* and *COL4A5*. After mutagenesis, sequences were verified. The *COL4A3/A4/A5* tagged constructs were subsequently cotransfected into human embryonic kidney 293 (HEK293T) cells. Twenty-four hours posttransfection, cells were replated in LumiNunc 96-well white plates (Thermo Fisher Scientific) and cultured in phenol red-free Dulbecco’s Modified Eagle Medium (DMEM) containing 10% fetal bovine serum, 100 U of penicillin and streptomycin, 2 mmol/L of glutamine, and 200 μmol/L of l-ascorbic acid 2-phosphate trisodium salt. After 24 hours, cells (intracellular heterotrimer) and media (secreted heterotrimer) were assayed using Nano-Glo Live Cell Assay reagent and GloMax Navigator system (Promega).

## Results

### Computational Validation

We identified 70 SNVs in *COL4A3* (29), *COL4A4* (26), and *COL4A5* (15) across 186 adults with FSGS with whole-exome sequencing. Of these, 31 were rare (minor allele frequency < 0.005), of which 30 were missense (14 in *COL4A3*, 10 in *COL4A4*, and 6 in *COL4A5*) and 1 was a stop-gain in *COL4A4*. Characteristics of the sequenced cohort have been published previously.^3^

In parallel, 2,803 nonsynonymous *COL4A3*/*A4*/*A5* variants were identified in 125,748 unscreened participants with whole-exome data in gnomAD, a public database of genomic variation. Of these, 2,307 were rare and 2,279 were missense variants.

**Figure 1:**
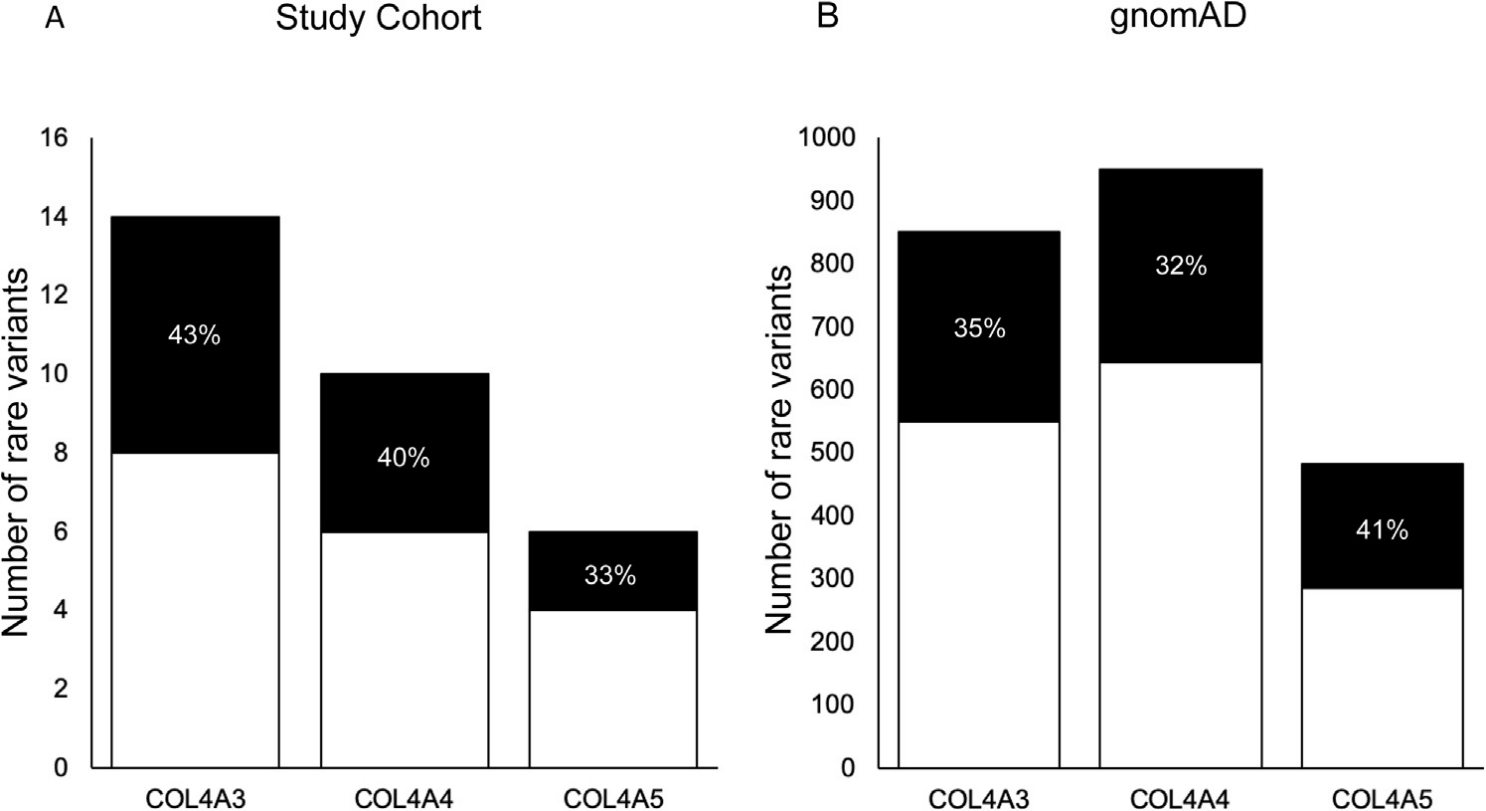
Number of rare missense variants predicted to be pathogenic in: (A) the focal segmental glomerulosclerosis (FSGS) study cohort and (B) Genome Aggregation Database (gnomAD). For rare missense *COL4A3*, *COL4A4*, and *COL4A5* variants in our FSGS cohort, 43% (6/14), 40% (4/10), and 33% (2/6) were predicted to be deleterious by at least 10 of 12 programs, respectively. For rare missense *COL4A3*, *COL4A4*, and *COL4A5* variants identified in gnomAD, 35% (301/851), 32% (306/949), and 41% (197/483) were predicted to be deleterious by at least 10 of 12 programs, respectively.

Rare missense variants in our local FSGS cohort, gnomAD, and Alport databases were each interrogated using 12 in silico programs for predictions (Table S1; Item S1). In the FSGS cohort, for rare missense variants in *COL4A3*, *COL4A4*, and *COL4A5*, 43% (6/14), 40% (4/10), and 33% (2/6) were predicted to be deleterious by at least 10 of 12 programs, respectively (Fig 1; Item S1).

By comparison, for rare missense variants in *COL4A3*, *COL4A4*, and *COL4A5* identified in gnomAD, 35% (301/851), 32% (306/949), and 41% (197/483) were predicted to be deleterious by at least 10 of 12 programs, respectively (Fig 1). gnomAD is a database in which some rare diseases would be even less represented than population estimates given that severe pediatric cases are not included.^48^ However, the lack of clinical data to correlate rare variants in gnomAD controls does not enable us to draw conclusions as to the accuracy of these predictions.

**Figure 2:**
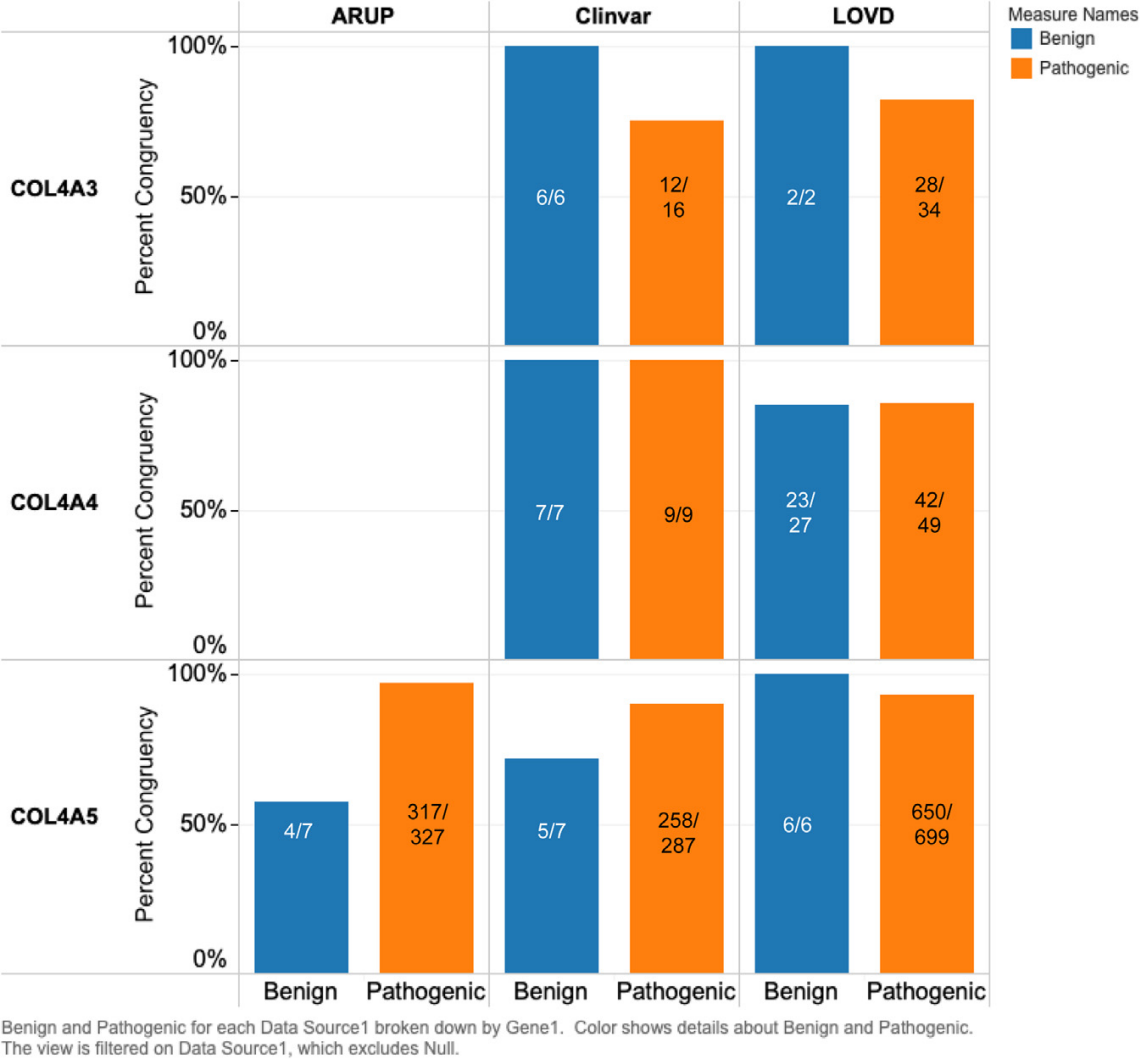
Comparison of *COL4A3*, *COL4A4*, and *COL4A5* in silico predictions with disease database categorization. For ARUP *COL4A5* pathogenic variants, there was 97% (317/327) concordance with in silico predictions. For ClinVar *COL4A3/A4/A5* pathogenic variants, there was 75% (12/16), 100% (9/9), and 89% (258/287) concordance with in silico predictions, respectively. For LOVD *COL4A3/A4/A5* pathogenic variants, there was 82% (28/34), 86% (42/49), and 94% (650/699) concordance. Congruency of in silico predictions was similar for variants categorized as benign, with the exception of *COL4A5* variants documented in ARUP and ClinVar, in which the effects were overestimated by in silico programs, though there were fewer variants to interrogate. In ARUP, 57% (4/7) of *COL4A5* variants were classified correctly by in silico predictions. In ClinVar, 100% (6/6), 100% (9/9), and 71% (5/7) of *COL4A3/A4/A5* variants, respectively, were correctly assigned. Finally, for LOVD, 100% (2/2), 85% (23/37), and 100% (6/6) of *COL4A3/A4/A5* variants were correctly classified.

We also accessed disease databases in which *COL4A3*, *COL4A4*, and *COL4A5* variants would be deposited, which included ARUP, ClinVar, and LOVD (accessed October 22, 2019, September 13, 2019, and August 28, 2019, respectively; Fig 2). ARUP documented 346 SNVs in *COL4A5*. Three hundred twenty-seven were categorized as pathogenic, with 97% (317/327) concordance to in silico predictions. ARUP does not document SNVs in *COL4A3* or *COL4A4*. In ClinVar, 120 *COL4A3* SNVs were documented. Sixteen were categorized as pathogenic and 75% were assigned (12/16) correctly by in silico programs. For *COL4A4*, 55 SNVs were reported. Nine were classified as pathogenic, with 100% assigned correctly by in silico programs. For *COL4A5*, there were 367 SNVs. Two hundred eighty-seven were categorized as pathogenic, with 90% (258/287) concordance to in silico predictions. In LOVD, 412 *COL4A3* SNVs were catalogued. Of these, 34 were pathogenic and 82% (28/34) were predicted accurately. For *COL4A4*, there were 306 SNVs, of which 49 were classified as pathogenic, with 86% (42/49) concordance to in silico predictions. For *COL4A5*, there were 987 SNVs. Six hundred ninety-nine were classified as pathogenic, with 94% (650/699) concordant in silico predictions. In silico program sensitivity could be overestimated given that they may have been used in the categorization of variants labeled as pathogenic in these disease databases.

The congruency of in silico predictions was similar for variants categorized as benign, with the exception of *COL4A5* variants documented in ARUP and ClinVar, in which the effects were overestimated by in silico programs, though there were fewer variants to interrogate (Fig 2). In ARUP, 7 *COL4A5* variants were classified as benign, 57% (4/7) of which were assigned as such by in silico predictions. In ClinVar, 6 *COL4A3*, 7 *COL4A4*, and 7 *COL4A5* variants were classified as benign, with 100%, 100%, and 71% (5/7) concordance with predictions, respectively. In LOVD, 2 *COL4A3* and 6 *COL4A5* variants were classified as benign, with 100% concordance for both. For *COL4A4*, there were 27 benign variants, with 85% (23/27) concordance with predictions.

A report suggests that one in silico classifier called M-CAP outperforms popular scores such as SIFT, PolyPhen-2, and CADD in its ability to separate pathogenic from benign variants.^45^ As a result, analysis of variants from disease databases was performed using M-CAP only. None of the benign variants in the disease databases were correctly classified, either as a result of incorrect categorization as pathogenic or by not generating an output (Fig S1). The accuracy for classification of pathogenic variants was much better, ranging from 89% to 96%. Additionally, a receiver operating curve for each of the 12 in silico programs was generated using the disease database type IV collagen variants and their in silico scores (Fig S2). When each curve is examined, we find that the score cutoff that maximizes the true-positive rate while minimizing the false-positive rate does not coincide with the in silico programs’ recommendations. For instance, we find that the cutoff for SIFT should be approximately less than 0.004, whereas the recommended cutoff is less than 0.05 (Fig S2). As a result, variants with scores between 0.004 and 0.05 are being predicted as pathogenic, leading to false positives.

**Figure 3:**
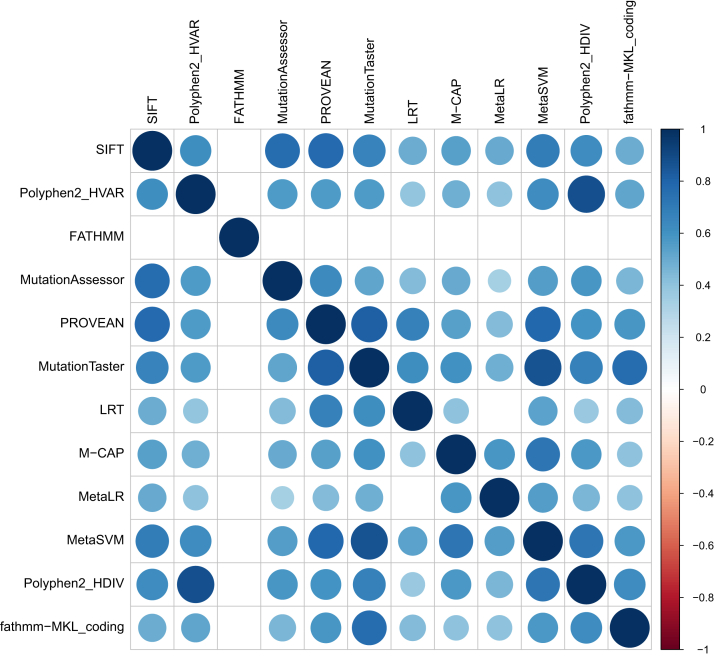
Spearman correlation coefficient heatmap comparing results of various prediction models. Most programs had similar prediction scores when comparing with each other except for FATHMM and M-CAP. Dark blue signifies a strong direct correlation while dark red signifies a strong indirect correlation. Squares that are lighter in color signify a weak correlation between the results of the 2 prediction models. Figure created using the corrplot package available in Rstudio.

Congruency in classification between in silico programs was also explored (Fig 3). Most programs had similar prediction scores when comparing with each other except for FATHMM and M-CAP.

### Functional Validation

**Figure 4:**
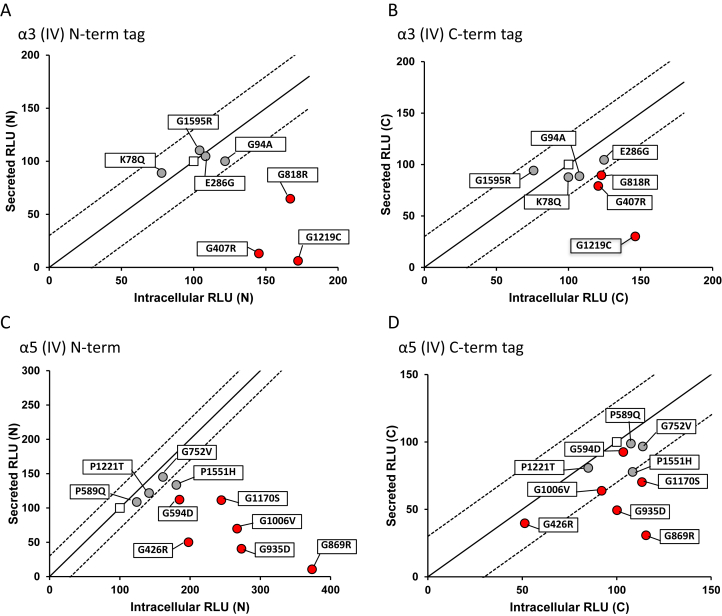
Functional characterization of *COL4A3* and *COL4A5* using the split-luciferase assay. Scatterplots of the intracellular/secreted relative light unit (RLU) ratio from human embryonic kidney 293 (HEK293T) cells expressing (A, B) mutant *α3* chain or (C, D) mutant *α5* chain compared to wild type (WT) using N-terminal and C-terminal split-luciferase tagged constructs. Pathogenic *α3* and *α5* chain mutants showed clearer secretory defect with N-terminal tagged constructs. Solid line: Y = X, dotted line: Y = X + 50, Y = X – 50. Square, WT; red circles are pathogenic variants and grey circles are variants of uncertain significance. Any data point under the −50 line was considered as a significant secretory defect. Experiments were performed in triplicate. Data presented are representative of 2 independent experiments.

We evaluated 9 pathogenic missense variants in *COL4A3* and the X-linked *COL4A5* identified in the local FSGS cohort. For *COL4A3* and *COL4A4*, the mode of inheritance has traditionally been reported as recessive, but next-generation sequencing studies have reported about 20% to 30% of patients with dominant disease.^49^, ^50^, ^51^, ^52^ Of 3 pathogenic heterozygous missense variants in *COL4A3* (ie, rare variant reported in other individuals with kidney disease), all were predicted to be deleterious by at least 10 of 12 in silico programs (Table 1; Item S2). Of 6 pathogenic variants in *COL4A5*, all were predicted to be deleterious by at least 10 of 12 in silico programs (Table 1). Under normal conditions, *COL4A3*, *COL4A4*, and *COL4A5* each encodes a protein that heterotrimerizes and is secreted into the glomerular basement membrane. To determine the secretory behavior of the *COL4A*3 and *COL4A*5 mutants, we used an assay system that quantified the intracellular trimerization and trimer secretion of *COL4A3/4/5*.^47^ Using this split luciferase complementation assay, all *COL4A3* and *COL4A5* pathogenic variants were found to have secretory defect with the N-terminal tagged versions of *COL4A3* and *COL4A5* (Fig 4A and C). The pathogenic variants could form trimers intracellularly but could not be efficiently secreted. By contrast, this was not always observed for C-terminal tagged versions of *COL4A3* and *COL4A5* (Fig 4B and D). We speculate that this could be due to heterotrimer formation being initiated at the noncollagenous (NC1) domain of the C-terminal region of collagen and terminates at the N-terminal region. The fusion of the monomers initially at the C-terminal region brings the reporter tags closer together to produce luminesce regardless of whether the trimer is completely formed.

**Table 1:** Comparison of Functional Annotation With In Silico Predictions for Pathogenic *COL4A3* and *COL4A5* Variants Identified in the FSGS Cohort

Gene	No. of Pathogenic Variants	No. Predicted Deleterious by 10/12 Programs	No. of Variants With Secretory Defect	Congruence
*COL4A3*	3	3	3	100%
*COL4A5*	6	6	6	100%

*Note:* All pathogenic *COL4A3* and *COL4A5* variants were categorized as such as a result of being identified in other kidney disease cases. All pathogenic variants demonstrated a secretory defect with functional characterization and were correctly assigned by in silico predictions.

Abbreviation: FSGS, focal segmental glomerulosclerosis.

Similarly, 8 variants of uncertain significance in *COL4A3* and *COL4A5* identified in our FSGS cohort were selected, comparing in silico predictions with functional characterization. Of 4 variants of uncertain significance in *COL4A3*, 3 were predicted to be deleterious by at least 10 of 12 in silico programs (Table 1). Of 4 variants of uncertain significance in *COL4A5*, 2 were predicted to be deleterious by at least 10 of 12 in silico programs (Table 1). Using the split luciferase complementation assay, only 1 variant of uncertain significance in *COL4A5* was found to have a secretory defect using N-terminal tagged proteins, though not to the degree observed for the definitely pathogenic variants (Fig 4C). Any data point under the −50 line was considered as a significant secretory defect. Thus, there was poor concordance between in silico predictions and functional characterization, with the former potentially overestimating the functional characteristics of missense variants (Table 2).

**Table 2:** Comparison of Functional Annotation With In Silico Predictions for Variants of Uncertain Significance in *COL4A3* and *COL4A5* Identified in the FSGS Cohort

Gene	No. of Variants of Uncertain Significance	No. Predicted Deleterious by 10/12 Programs	No. of Variants With Secretory Defect	Congruence
*COL4A3*	4	3	0	0%
*COL4A5*	4	2	1	50%

*Note:* Only 1 variant of uncertain significance in *COL4A5* was found to have a secretory defect, which was accurately predicted by in silico predictions. However, 1 variant of uncertain significance without evidence of a secretory defect was also predicted to be deleterious.

Abbreviation: FSGS, focal segmental glomerulosclerosis.

To further determine the functional nature of 8 variants of uncertain significance in *COL4A3* and *COL4A5*, clinical characteristics for these patients were obtained (Tables S2 and S3). Many individuals with Alport syndrome have hematuria and basement membrane abnormalities. In our cohort, microscopic hematuria data were reported for 9 patients with pathogenic variants and 8 patients with a variant of uncertain significance in *COL4A3* and *COL4A5*. Microscopic hematuria was observed in 4 of 9 patients with pathogenic variants and 2 of 8 patients with a variant of uncertain significance (Tables S2 and S3). For the 2 patients with variants of uncertain significance (*COL4A3* p.G1595R and *COL4A5* p.P589Q) and hematuria, neither variant was characterized as functionally deleterious (Fig 4).

## Discussion

Our results demonstrate that in silico predictions correctly classified most pathogenic *COL4A3/A4/A5* variants catalogued in ClinVar, ARUP, and LOVD. In silico predictions performed similarly for benign variants with the exception of *COL4A5* (concordance in ARUP and ClinVar with predictions and classification was 57% [4/7] and 71% [5/7], respectively) but there were also far fewer benign variants to interrogate in these disease databases. Our second approach of correlating in silico predictions with functional testing showed that both accurately classified all pathogenic *COL4A3/A4/A5* missense variants in the FSGS cohort. These variants were labeled as pathogenic because they are rare and already reported as disease-causing in other individuals with kidney disease, which are considered strong lines of evidence (ACMG criteria PS1 and PS4; Item S2).^11^ By contrast, in silico predictions overestimated the effects of *COL4A3/A5* variants of uncertain significance when compared with functional characterization. A variant of uncertain significance was defined as a rare variant that did not satisfy ACMG criteria for definite pathogenicity. Interestingly, interrogation of *COL4A3/A4/A5* variants found in gnomAD predicted a high percentage to be deleterious, but the lack of clinical data for correlation prevents us from making any conclusion with these data.

Genomics facilitates clinically meaningful classification of CKD but sequencing can reveal rare SNVs for which the relationship to disease is unclear. The ACMG has standards based on expert consensus for declaring pathogenicity wherein in silico predictions are considered only supporting compared with higher levels of evidence that are deemed moderate, strong, or very strong.^11^ Well-established functional studies that show deleterious effect is an example of one criterion considered strong level of evidence. Against this background, we provide an assessment of in silico programs using both computational and experimental approaches.

Using the Nano-luciferase complementation system, we have recently quantified trimerization of 9 typical glycine substitutions in *COL4A5* that differ in disease progression, finding a correlation between in vitro results and phenotype.^53^ In the data presented here, we observe that the pattern of heterotrimer formation and secretion for pathogenic mutants differed slightly between N-terminal and C-terminal tagged constructs. The N-terminal tagged pathogenic mutants showed clearer secretory defect. We postulate that this could be as a result of heterotrimer formation initiating at the NC1 domain at the C-terminal region of collagen and terminates at the N-terminal region. The fusion of the monomers initially at the C-terminal region could bring the reporter tags closer together to produce luminesce regardless of whether the heterotrimer is completely formed. Therefore, the luciferase reporter attached at the N-terminal region, that is, the N-tagged constructs, may better reflect the state of trimer folding.

The 12 prediction models used in this study can be categorized as solely conservation based: (SIFT Polyphen2-HVAR, Polyphen2-HDIV, MutationAssessor, PROVEAN, and LRT) and multifeatured algorithms (FATHMM, M-CAP, MetaLR, MetaSVM, FATHMM-MKL, MutationTaster; Table S1). Conservation-based models select homologous sequences to create multiple sequence alignments across species (MSA) and use the sequence and predicted structure-based features of the MSA to predict pathogenicity with variants in more conserved areas predicted to be deleterious. The multifeatured algorithms integrate other information, such as epigenomic signals (FATHMM- MKL and MutationTaster), allele frequencies (FATHMM, MetaLR, and MetaSVM), or the results of other prediction algorithms (M-CAP, MetaLR, and MetaSVM). Eleven of the 12 prediction models are trained using databases including UniProt^54^ (PolyPhen2-HDIV, PolyPhen2-HVAR, FATHMM, PROVEAN, MetaLR, and MetaSVM), Human Gene Mutation Database^55^ (FATHMM, FATHMM-MKL, MutationTaster, and M-CAP), ExAC^56^ (M-CAP), Ensembl^57^ (LRT), 1000 Genomes Project^58^ (MutationTaster and FATHMM-MKL), and COSMIC^59^ (MutationAssessor). SIFT was trained using known variants of the *E coli* LacI gene that have been individually mutated and functionally tested.^60^^,^^61^

M-CAP has been previously reported to outperform popular pathogenicity classifiers but our results demonstrate that it unreliably categorizes the small number of benign type IV collagen variants in disease databases by incorrectly assigning pathogenicity or not generating an output. M-CAP already uses 9 established pathogenicity likelihood scores included in our scoring system: SIFT13, PolyPhen-2, CADD15, MutationTaster20, MutationAssessor21, FATHMM22, LRT23, MetaLR16, and MetaSVM16.^45^ It incorporates 7 established measures of base pair, amino acid, genomic region, and gene conservation: RVIS24, PhyloP25, PhastCons26, PAM250, BLOSUM62, SIPHY28, and GERP29. Additionally, M-CAP introduces 298 new features derived from multiple-sequence alignment of 99 primate, mammalian, and vertebrate genomes to the human genome30. However, previous reports seeking to demonstrate superiority of one classifier over others are all limited by the veracity of variant assignment in test databases and in which kidney gene variants contribute a small proportion.

Our study highlights several limitations and opportunities for future investigation. Estimating in silico program accuracy using disease databases relies on robust categorization and underscores a need for consistency in variant annotation. The sensitivity of in silico programs could be overestimated given that they may have been used in the categorization of variants labeled as pathogenic. In disease databases, there were far fewer variants classified as benign compared with pathogenic. However, to address these limitations, we have pursued more laborious functional characterization on randomly selected type IV collagen variants from the FSGS cohort as an additional line of evidence.

With respect to functional characterization, we include data to support our conclusions, but only a small number of missense variants were characterized. We use the arbitrary cutoff of ±50 from wild-type data, but characterizing more pathogenic and benign variants would better define a threshold. As per standard convention throughout the literature, we characterize the effects of single variants on the reference haplotype, but there are several common haplotypes documented in the 1000 Genomes Project (Table S4). Studying the effects of single variants on different haplotype backgrounds could provide important information regarding interaction effect between haplotype and mutation. Second, our assay will identify mutations that are associated with secretory defects, but this is a simplification of disease pathogenesis that does not account for the complexities involving extracellular type IV collagen network formation. For instance, a previous report suggests that ∼20% of *COL4A5* mutations have detectable heterotrimers in the glomerular basement membrane, suggesting alternate disease mechanisms.^62^

Recent reports demonstrate that pathogenic variants in *COL4A3*/*A4*/*A5* account for a significant and unappreciated proportion of patients with Alport syndrome in CKD.^3^^,^^5^, ^6^, ^7^, ^8^, ^9^, ^10^^,^^63^ Sequencing is increasingly being used to obtain mechanistically relevant diagnoses but often generates rare missense variants that remain of uncertain clinical significance. In silico predictions have been developed to aid in categorizing variants. We show here that computational approaches including M-CAP, which was reported to outperform other classifiers, are sensitive but not sufficiently specific to confidently assign *COL4A3*/*A4*/*A5* variant pathogenicity. Thus, we do not recommend any in silico program in the consideration of type IV collagen variant categorization, but instead pursuing more objective levels of evidence suggested by medical genetic guidelines.

## Supplementary Material

Supplementary File 1 (PDF)

Supplementary File 2 (XLS)

Supplementary File 3 (XLS)
