# Supplementary material for: Type IV Collagen Variants in CKD: Performance of Computational Predictions for Identifying Pathogenic Variants
Source: Kidney Med. 2021 Feb 10;3(2):257–66. doi: 10.1016/j.xkme.2020.12.007 (PMC8039416; doi:10.1016/j.xkme.2020.12.007)
Supplement: Supplementary File 1 (PDF) [file mmc1.pdf]

Table S1. Twelve *In Silico* Predictions Programs Used.

**Program**	**Method**	**Training Database**	**Testing Database**	**Information used for model**
SIFT	Position-specific scoring matrix	E. Coli LacI gene	HIV-1 protease and bacteriophage T4 lysozyme genes	Sequence homology<br>https://www.ncbi.nlm.nih.gov/pmc/articles/PMC311071/
PolyPhen2-HDIV	Naïve Bayes Classifier	UniProt<br><br>HumDiv is Mendelian disease variants vs. divergence from close mammalian homologs of human proteins (>=95% sequence identity).	UniProt	8 Sequence-based features<br>- PSIC score<br>- Sequence identity<br>- CgG context<br>- Congruency to MSA<br>- Probability of substitution based on congruency<br>- Alignment depth<br>- Change in amino acid volume<br>- Location in Pfam domain<br>3 Structure-based features<br>- Accessible surface area of wild-type amino acid residue<br>- Change in hydrophobic propensity<br>- Conformational mobility<br>https://www.ncbi.nlm.nih.gov/pmc/articles/PMC2855889/#SD1<br>https://www.ncbi.nlm.nih.gov/pmc/articles/PMC4480630/
PolyPhen2-HVAR	Naïve Bayes Classifier	UniProt; HumVar is all human variants associated with some disease (except cancer mutations) or loss of activity/function vs. common (MAF>1%) human polymorphism with no reported association with a disease of other effect	UniProt	Same as above

Program	Method	Training Database	Testing Database	Information used for model
FATHMM	Hidden Markov models	HGMD + UniProt	VariBench + literature +SwissVar portal	Sequence homology + relative frequency of disease-associated and functionally neutral amino acid substitutions<br><br>https://www.ncbi.nlm.nih.gov/pmc/articles/PMC3558800/
FATHMM-MKL	Multiple kernel learning	HGMD + 1000G	HGMD + 1000G + ClinVar	10 feature groups:<br>- 46-Way Sequence Conservation<br>- Histone Modifications (ChIP-Seq)<br>- Transcription Factor Binding Sites (TFBS PeakSeq)<br>- Open Chromatin (DNase-Seq)<br>- 100-Way Sequence Conservation<br>- GC Content<br>- Open Chromatin (FAIRE)<br>- Transcription Factor Binding Sites (TFBS SPP)<br>- Genome Segmentation<br>- ENCODE annotations<br><br>https://academic.oup.com/bioinformatics/article/31/10/1536/177080
MutationAssessor	Combinatorial entropy optimization	COSMIC	COSMIC	Sequence homology + specificity residues between subfamilies<br><br>http://mutationassessor.org/r3/MutationAssessor_white_paper.pdf
PROVEAN	Alignment score	UniProt/ HUMSAVAR	UniProt + Swiss-Prot + TP53 genes + ABCA1 genes	Sequence homology<br><br>https://journals.plos.org/plosone/article?id=10.1371/journal.pone.0046688
MutationTaster	Bayes classifier	HGMD + 1000G	HGMD + 1000G + ClinVar	Conservation, splice site, mRNA features, protein features, allele frequencies from HGMD and 1000G<br><br>http://www.mutationtaster.org/info/documentation.html
LRT	Likelihood ratio test	32 vertebrate genomes	OMIM database + literature + 3 genomes (Venter, Watson, and Chinese genome)	Sequence homology<br><br>https://www.ncbi.nlm.nih.gov/pmc/articles/PMC2752137/

Program	Method	Training Database	Testing Database	Information used for model
M-CAP	Gradient boosting tree classifier	Rare variants in HGMD + ExAC	Rare variants in HGMD + ExAC	9 pathogenicity likelihood scores<br>- SIFT<br>- PolyPhen2<br>- CADD<br>- MutationTaster<br>- MutationAssessor<br>- FATHMM<br>- LRT<br>- MetaLR<br>- MetaSVM<br>7 measures of genetic conservation<br>- RVIS<br>- PhyloP<br>- PhastCons<br>- PAM250<br>- BLOSUM62<br>- SIPHY<br>- GERP<br>298 new features derived from primate, mammalian, and vertebrate genomes MSA*
MetaLR	Logistic regression	UniProt	Literature + CHARGE + VariBench	Nine prediction scores<br>- PolyPhen-2<br>- SIFT<br>- MutationTaster<br>- Mutation Assessor<br>- FATHMM<br>- LRT<br>- GERP++<br>- SiPhy<br>- PhyloP<br>Allele frequencies in 1000G<br>https://www.ncbi.nlm.nih.gov/pmc/articles/PMC4375422/
MetaSVM	Support vector machine	UniProt	Literature + CHARGE + VariBench	Same as above<br>https://www.ncbi.nlm.nih.gov/pmc/articles/PMC4375422/

*https://www.nature.com/articles/ng.3703.epdf?author_access_token=QpYcDWHWC2sC08xquy51RtRgN0jAjWel9jnR3ZoTv0NW_LUqRr20bAF4DHicakv7aAze4axhulp3iw7_FiWtLJK80EJeraUYDseDG607CfszMjiK1pq2G7wodelPyGeQ

Programs and Access dates

SIFT (February 3, 2015 ensembl 66 version)[1], PolyPhen2-HDIV (April 11, 2012 version)[2, 3], Polyphen2- HVAR (April 11, 2012 version) [2, 3], FATHMM (August 13, 2015 version)[4], FATHMM-MKL (August 3, 2015 version) [5], MutationAssessor (March 20, 2016 release 3) [6, 7], MutationTaster (March 20, 2016 ensemble 69 version) [8], PROVEAN (February 3, 2015 score v1.1) [9, 10], LRT (October 3, 2013 version) [11], M-CAP (November 30, 2016 version) [12], MetaLR (January 26, 2014 version)[13], and MetaSVM January 26, 2014 version)[14].

Table S2. Clinical Characteristics of FSGS Patients with *COL4A3* Variants.

ID	Variant	Any Other Variant?	Sex	Age of Onset	Peak proteinuria (g)	Hematuria	Nadir serum albumin (g/L)	Treatment	Treatment Response	ESRD	GBM comments in biopsy report
COL4A3: Pathogenic											
6062	G407R (het)	No	F	30	4	No	unk	16 weeks of prednisone	Resistant	unk	Diffusely thin
7215	G818R (het)	No	F	13	unk	No	unk	prednisone, tacrolimus and MMF	Resistant	No	Abnormal
2555	G1219C (het)	No	F	36	4.8	Microscopic hematuria	40	62 weeks of prednisone and azathioprine	Resistant	yes	Normal
COL4A3: Variant of Uncertain Significance											
6085	K78Q (het)	No	F	unk	unk	No	unk	unk	unk	unk	Normal
2564	G94A (het)	No	F	22	unk	No	unk	Steroids	Unknown response	No	Normal
7901	E286G (het)	No	M	5	2.1	No	39	Steroids	Partial response	No	Normal
2378	G1595R (het)	*INF2* R106P (het)	M	22	8.2	Microscopic hematuria	33	8 weeks of cyclosporine treatment	Resistant	yes	unk

Table S3. Clinical Characteristics of FSGS Patients with *COL4A5* Variants.

ID	Variant	Any Other Variant?	Sex	Age of onset	Peak proteinuria (g)	Hematuria	Nadir serum albumin (g/L)	Treatment	Treatment Response	ESRD	GBM comments in biopsy report
COL4A5: Pathogenic											
2594	G426R (het)	No	F	28	unk	Microscopic hematuria	35	Cyclosporine	Resistant	Yes	unk
4976	G594D (het)	No	M	41	7.58	No	42	Steroid	Resistant	No	Focally thin
6223	G869R (het)	No	F	unk	unk	No	unk	unk	Unknown response	Yes	unk
2480	G935D (het)	No	F	unk	unk	No	unk	Prednisone	Resistant	Yes	unk
1590	G1006V (het)	No	F	28	4.12	Microscopic hematuria	27	unk	Unknown response	No	unk
5269	G1170S (het)	No	M	57	7.19	Microscopic hematuria	26	Steroid and cyclosporine	Partial response	Yes	Irregular and focally thin
COL4A5: Variant of Uncertain Significance											
2555	P589Q (het)	No	F	36	4.8	Microscopic hematuria	40	Prednisone and azathioprine	Resistant	Yes	Normal
2738	G752V (het)	No	M	49	2.38	No	41	None	U/A	No	No EM

2690	P1221T (het)	No	M	56	11.15	No	36	unk	unk	Yes	Normal
7941	P1551H (het)	No	M	47	15	No	31	Prednisone and different immunosuppr essant	Partial response	No	Normal

ESRD = end-stage renal disease, GBM = glomerular basement membrane, unk = unknown, het=heterozygous
Family history only in 7215 reported but no other relatives available to test for segregation studies.

		Rs13424243				Rs10178458			Rs6436669				Rs55703767				Rs11677877				Rs28381984			
		G43R				L141P			E162G				D326Y				H451R				P574L			
exon #		2				7			9				17				22				25			
ref AA	...FCD	G	AKG...		...PGT	L	GYP...	...PAK	E	EDI...		...MGE	D	GIK...		...PGD	H	GLP...		...PGT	P	GVK...		
alt AA		R				P			G				Y				R				L			
ancestral allele		G				T			G				G				G				C			
contig allele		G				T			A				G				A				C			
major allele		G				C			G				G				A				C			
minor allele		C				T			A				T				G				T			
MAF		0.354				0.16			0.161				0.212				0.069				0.473			
1KG EUR haplotypes																							haplotype frequency	
H1		G				C			G				G				A				T		0.233	
H2		G				C			G				G				A				C		0.16	
H3		C				C			G				G				A				T		0.154	
H4		C				C			G				T				A				C		0.096	
H5		G				C			G				T				A				C		0.093	
H6		G				T			A				G				A				T		0.066	
H7		C				C			G				G				G				C		0.038	
H8		G				C			G				G				G				C		0.037	
H9		G				T			A				G				A				C		0.037	
...																								

Supplementary Table 4. Common *COL4A3* Haplotypes Identified in Europeans in the 1000 Genomes Project

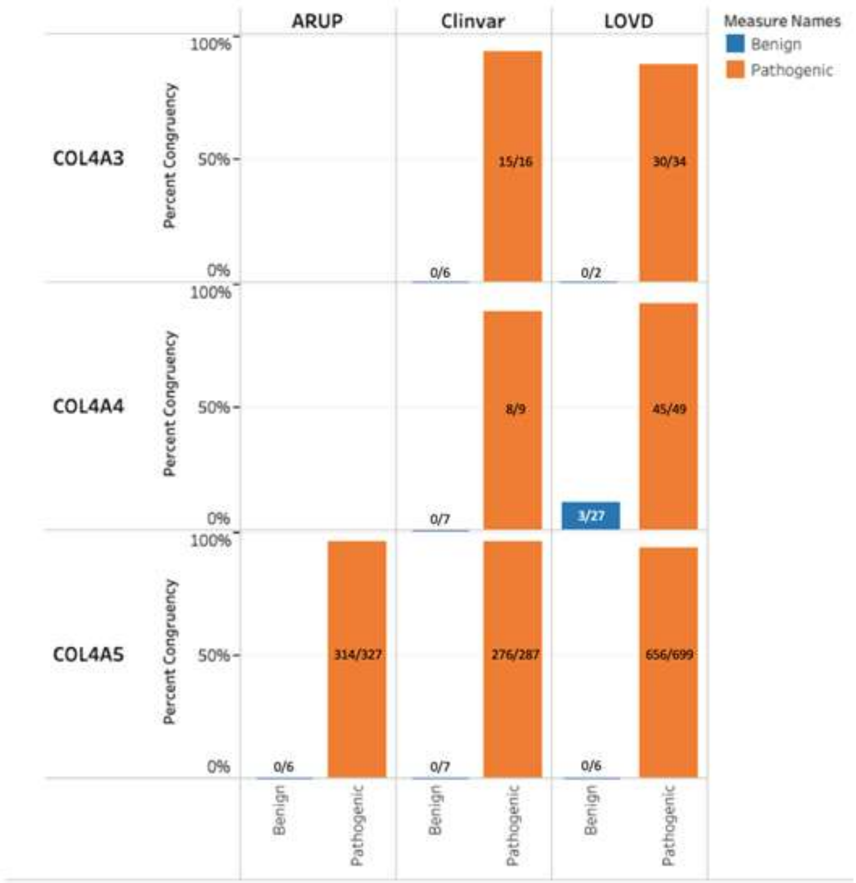

Supplementary Figure 1. Comparison of *COL4A3*, *COL4A4* and *COL4A5 in silico* predictions by M-CAP with disease database categorization. Shown are the total number of variants tested in each database.

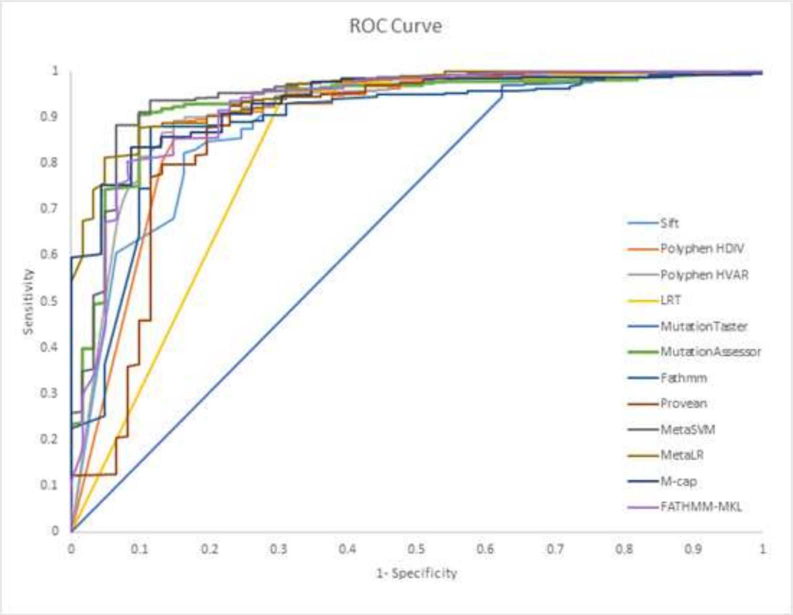

Program	Recommended cut-off	Our predicted cut-off for pathogenicity
SIFT	<0.05	<0.004
Polyphen-HDIV	>0.956	>0.99
Polyphen-HVAR	>0.909	>0.953
LRT	*	<0.001
MutationTaster	>0.5	>0.999
MutationAssessor	>3.5	>3.115
FATHMM	<-1.5	<-4.10
Provean	<-2.5	<-5.23
MetaSVM	>0.82268	>0.904
MetaLR	>0.5	>0.907
M-Cap	>0.025	>0.858
FATHMM-MKL	>0	>0.964

Supplementary Figure 2. Receiver operator curves for the 12 *in silico* programs using scores generated from type IV collagen variants obtained from disease databases. We find that the optimal cut-offs to maximize sensitivity while minimizing false positives (1-specificity) does not correlate with *in silico* program recommendations. * score is not only consideration in categorization of pathogenicity.

References

1. Ng, PC, Henikoff, S: SIFT: Predicting amino acid changes that affect protein function. *Nucleic Acids Research,* 31**:** 3812-3814, 2003.
2. Adzhubei, IA, Schmidt, S, Peshkin, L, Ramensky, VE, Gerasimova, A, Bork, P, Kondrashov, AS, Sunyaev, SR: A method and server for predicting damaging missense mutations. *Nature Methods.* NIH Public Access, 2010 pp 248-249.
3. Ramensky, V: Human non-synonymous SNPs: server and survey. *Nucleic Acids Research,* 30**:** 3894-3900, 2002.
4. Shihab, HA, Gough, J, Cooper, DN, Stenson, PD, Barker, GLA, Edwards, KJ, Day, INM, Gaunt, TR: Predicting the Functional, Molecular, and Phenotypic Consequences of Amino Acid Substitutions using Hidden Markov Models. *Human Mutation,* 34**:** 57-65, 2013.
5. Shihab, HA, Rogers, MF, Gough, J, Mort, M, Cooper, DN, Day, INM, Gaunt, TR, Campbell, C: An integrative approach to predicting the functional effects of non-coding and coding sequence variation. *Bioinformatics,* 31**:** 1536-1543, 2015.
6. Reva, B, Antipin, Y, Sander, C: Predicting the functional impact of protein mutations: Application to cancer genomics. *Nucleic Acids Research,* 39**:** e118-e118, 2011.
7. Reva, B, Antipin, Y, Sander, C: Determinants of protein function revealed by combinatorial entropy optimization. *Genome Biology,* 8**:** R232, 2007.
8. Schwarz, JM, Cooper, DN, Schuelke, M, Seelow, D: Mutationtaster2: Mutation prediction for the deep-sequencing age. *Nature Methods.* 2014 pp 361-362.
9. Choi, Y, Chan, AP: PROVEAN web server: A tool to predict the functional effect of amino acid substitutions and indels. *Bioinformatics,* 31**:** 2745-2747, 2015.
10. Choi, Y, Sims, GE, Murphy, S, Miller, JR, Chan, AP: Predicting the Functional Effect of Amino Acid Substitutions and Indels. *PLoS ONE,* 7**:** e46688, 2012.
11. Chun, S, Fay, JC: Identification of deleterious mutations within three human genomes. *Genome Research,* 19**:** 1553-1561, 2009.
12. Jagadeesh, KA, Wenger, AM, Berger, MJ, Guturu, H, Stenson, PD, Cooper, DN, Bernstein, JA, Bejerano, G: M-CAP eliminates a majority of variants of uncertain significance in clinical exomes at high sensitivity. *Nature Genetics,* 48**:** 1581-1586, 2016.
13. Dong, C, Wei, P, Jian, X, Gibbs, R, Boerwinkle, E, Wang, K, Liu, X: Comparison and integration of deleteriousness prediction methods for nonsynonymous SNVs in whole exome sequencing studies. *Human Molecular Genetics,* 24**:** 2125-2137, 2015.
14. Kim, S, Jhong, JH, Lee, J, Koo, JY: Meta-analytic support vector machine for integrating multiple omics data. *BioData Mining,* 10**:** 2, 2017.
